# Gleanings from Our Exchanges

**Published:** 1872-07-01

**Authors:** 


### Gleanings from Our Exchanges.

Surgical Treatment of Aneurism.—The lectures of Mr. Holmes before the Royal College of Surgeons of England were devoted to this subject. The main propositions sustained by the accomplished lecturer were as follows:

1. Aneurisms, of whatever form or however near the heart they may be, ought not to be regarded as incurable, but should be made the objects of definite methodical treatment, internal or external.

2. There is definite proof, from pathological anatomy and from surgical experience, of the curative influence of Brasdor's operation in innominate aneurism, and of its beneficial effects in some cases of aortic aneurism.

3. Arteries may be successfully tied and obliterated without their continuity being interrupted; and this modification of the ligature, whilst affording much security against secondary hæmorrhage, and thus much diminishing the danger of the operation in general, may very probably in future enable surgeons to deal successfully with cases in which it may be necessary to tie the first part of the subclavian (whether on the distal or the proximal side of an aneurism) or the innominate artery.

4. Galvano-puncture may be used with, at any rate, temporary benefit in thoracic aneurism; its use is not so dangerous as to render further trials of it inexpedient; and there is good hope that the method may be so far perfected as to make it a safe and regular plan for the treatment of thoracic, subclavian, and other forms of aneurism.

5. Many cases, such as those in which ligature of the artery near to the heart has been resorted to for the cure of subclavian and subclavio-axillary aneurism, may be made amenable to improved methods of pressure.

6. Aneurismal tumors situated even as high as the lower part of the abdominal aorta, those of the mesenteric and other branches of the aorta, and of the iliac arteries, may be treated with success by rapid coagulation of blood under pressure; but this method is a dangerous one, and should not be used until internal treatment has failed.

7. There are cases of abdominal aneurism in which Mr. Syme's suggestion of reviving the old operation is worthy of further trial. —*The Doctor.*

Diagnosis of the First Stage of Carcinoma Colli Sterni.—Prof. Otto Spielberg, speaking of the difficulty of distinguishing between simple inflammatory induration of the cervix uteri, and carcinomatous infiltration, gives the following as a certain indication of cancerous infiltration, viz.: "*A peculiar induration of the cervix, the disposition of its mucous membrane, and its reaction to the dilatation of sponge tents.*" He expounds each member of this rule.

The hardness of cancerous deposit in comparison with simple induration, is well known; but the distinction is frequently impossible to make out, even by the most cultivated touch. The two other symptoms are unequivocal, and are as follows:

"First, the mucous membrane in cancerous growth is firmly connected with the underlying induration, and immovable over it, which is not the case in mere hyperplastic thickening and induration; and, second, while the latter, under the pressure of compressed sponge, in the cervical canal, becomes regularly even, though at times inconsiderably looser, softer and thinner, the cancerous infiltration remains unalterably hard and rigid, and cannot be stretched." He goes on to explain the reason for this difference between the products of the two inflammations from the locality where the cancerous inflammation originates, which is the utero-malphigii, or, in extremely rare cases, from the glands of the cervical canal. The latter form gives rise to the alveolar or colloid form, of which he has only seen one case. As a rule, the disease is developed from the interpapillary depressions of the epithelium. According as the growth of the epithelium into the tissues below is or is not attended by a simultaneous growth of the papillæ, two forms of cancer may be distinguished—the papillary velous, or cauliflower excrescence, and the simple infiltrated form.—*Cincinnati Clinic* (*from Archiv fur Gynækologie*).

---

Scholmann on Sulphate of Quinine in Cholera.—In the *All. Wien. Med. Zeit.*, Feb., 1872, Dr. Scholmann says that in 1866 several American physicians, in the cholera epidemic of the Mississippi Valley, had obtained remarkable results from sulphate of quinine. The author lost, in the epidemic of 1866, in Western Texas, out of 220 cases of choleraic diarrhœa and cholerine only three cases when he used the sulphate of quinine in gramme doses every two or three hours. Occasionally a quarter-grain dose of morphia was subcutaneously injected. When the diarrhœa did not disappear quickly, small doses of calomel were given hourly. Rest in bed and careful diet were observed. The author looks upon the way in which quinine acts as being due to its antiseptic properties; and he looks upon cholera, not as a parasitic disease, but as an acute tonic gastro-enteritis, with which is conjoined, in the period of asphyxia, an universal spasm of the vessels of the most marked form, a tetanus of the arteries. In whatever manner the poisonous particles enter the system is immaterial; the first symptoms of their entry are confined to the mucous membrane of the intestines, and it appears probable that at first the cause of the disease is a local and distinct one. A slight incubation precedes the disease, on which the catarrhal stage follows.—*Medical World.*

---

On the Treatment of Diabetes by Lactic Acid (Cantani's Method).—Dr. G. W. Balfour reports a series of cases of diabetes treated by Cantani's method with marked success. This method, as is well known, consists first, in confining the patient to an exclusively meat diet. This means plain meat roasted or boiled, without any sauces of milk or eggs, and certainly without any bread, flour, or any vegetable matter whatever, the only seasoning permitted being salt, oil, and a little vinegar. For drink he allows water, either plain or with a little of the purest alcohol; coffee, tea and wine being prohibited. The medicinal treatment being lactic acid 77 to 154 grains daily, diluted in from 8 to 10 fluid ounces of water. The lactic acid used by Dr. Balfour was obtained from the druggist fluid, not syrupy, of spec. grav. of 1.027, and with the ordinary smell of sour milk. The first case which the Dr. reports was complicated with phthisis. The patient died after about three months' treatment, having had no sugar in the urine for some time. The fifth case was much benefitted by the treatment. The sugar had entirely disappeared from the urine but he was disorderly and discharged from the hospital. Case 3, female, aged 15; Case 6, female, aged 17; case 4, male, aged 25; case 7, male, aged 35, had all been suffering from the disease from five months to three or four years. They are all much improved in strength and weight, and have no sugar in the urine, or at most only occasional traces of it; they are, however, still under observation. Case 2, male, aged 53; had been under treatment for the disease for more than a year. After three months' treatment, having for some time been restored to general diet, and no sugar appearing in the urine, he was pronounced cured and discharged. He has continued well since.

---

Dr. Bonfigli's Summary of Therapeutics.—In the *Riv. Clin. di Bologna*, February, 1872, Dr. Bonfigli mentions that Jones recommends belladonna in gradually increasing doses in spermatorrhœa. Atropine is recommended by Hosch in many cases of myopia. Philipson recommends belladonna in constipation.

Echeverria and MacDonald recommend conium as the best narcotic remedy for epileptics. The juice of the fresh plant is used.

Mr. Veagh considers datura tatulæ the best remedy for asthma, far better than stramonium.

Dr. Reichard, of Riga, has found chloral hydrate of great use in cholera; four grammes of chloral produced sleep in a few minutes, and the patient recovered.

Dr. Lutz has found great benefit from the use of bromide of potassium in epilepsy. His doses are large, from 10 to 20 grammes daily. No great inconvenience has been produced, except some few papules on the skin. In one case of nocturnal incontinence of urine it was very useful. Dr. Northrop says that a gramme of this salt every hour will get rid of tapeworm.

Dr. Palmberg has used ergot of rye with success in three cases of chronic diarrhœa. To children he gave 50 centigramme doses of the ergot in watery extract in one day; to adults he gives 1.20 grammes.

Dr. Vance alleges that he has obtained cures in some cases of palsy by the subcutaneous injection of strychnine, in the dose of five millegrammes of the phosphate.

---

Chloralum and Preparations of Chloralum as Disinfectants.—Prof. A. Fleck, Director of the Central Chemical Institution established at Dresden for the protection of the public health, has made a thorough investigation of the composition and value of these products, and with the following results:

1. The preparations of chloralum have nothing in common with the similarly sounding chloral hydrate, and are, in point of fact, mixtures of chloride of aluminium.

2. The preparations of chloralum contain chlorine combinations of lead, copper and arsenic, which renders their employment not free from danger, and which would render their employment as a medicine or as an astringent for open or suppurating wounds dangerous.

3. The price of the preparations of chloralum bears no relation either to their nature or their effect. Considering that the liquid chloralum yields a clear profit of at least 700 per cent., and the wadding 400 per cent., the limits of honest trading may be considered as overstepped.

4. The result of these experiments is that chloralum and the preparations made from the same must be classed among the worthless arcana, and in the interest of the public health, as well as in the material interests of the public, a most decided warning must be given against the purchase of the same.—*American Journal of Pharmacy, June*, 1871, *from Chemical Review, March*, 1872, *and Industrie Zeitung.*

Photographing the Pulse.—The ingenious apparatus invented by Dr. Ozanam, of Paris, for rendering the variable beatings of the pulse visible, is already proving itself of practical value. It consists of a camera lucida, about ten inches wide, in which a piece of mechanism, moving at a uniform rate, pushes a glass plate, prepared with collodion, in front of a very narrow aperture exposed to the light. In this aperture is a glass tube, in which a column of mercury may rise and fall, as in a thermometer. By attaching to the wrist a rubber tube, filled with mercury, in connection with the tube of the apparatus, the beating of the pulse is received on this artificial artery, and the pulsations are transmitted to the recording apparatus. As the column in the tube acts as a screen, light can penetrate the aperture only where the column is deficient; consequently the prepared plate becomes black under the influence of light everywhere except at such places as the column intercepts it. As the column rises and falls with each pulsation of the heart, these black lines on the prepared plate, pushed regularly forward, will be shorter or longer alternately, and will be successively photographed as being lines perpendicular to a common base, the heart being thus made to register photographically its own pulsations. These photographic representations can be so magnified as to be rendered visible across a large amphitheatre; and such is the peculiarity of the apparatus, in its adaption to different uses, that it may be modified so as to register the variations of respiration, the irregular action of coughing, and similar physiological and pathological phenomena.—*The Arts.*

---

Ozone Tests.—(1.) A strong phosphorous odor. (2.) A solution of iodide of potash and starch is colored blue by the same. (3.) A solution of thallic suboxide produces in a solution of ozone a brown precipitate of thallic oxide. Paper saturated with thallic suboxide is rendered brown by ozone. Paper saturated with salts is colored brown, that saturated with plumbic sulphuret or indigo is bleached. Blue litmus paper is bleached, turning finally yellowish red. An ozone solution, shaken with pure ethylic ether and potassic bichromate, does not color the ether blue.—*The Arts.*

---

Digitalis Infusion.—According to Richelot (*L'Union Med.*) the infusion of digitalis is more effective in dropsy than the decoction. Half-drachm to six ounces of water should be used in twenty-four hours.

